# Perforating Disseminated Necrobiosis Lipoidica Diabeticorum

**DOI:** 10.1155/2013/370361

**Published:** 2013-02-25

**Authors:** Paula Lozanova, Lyubomir Dourmishev, Snejina Vassileva, Ljubka Miteva, Maria Balabanova

**Affiliations:** Department of Dermatology and Venereology, Medical University Sofia, Sofia, Bulgaria

## Abstract

Perforating necrobiosis lipoidica is a very rare clinical variant which consists of degeneration and transepidermal elimination of the collagen with few cases reported in the literature. In two-thirds of the patients it associates with diabetes, with no relation with the glucose control. We present a 42-year-old female patient with a 7-year history of diabetes on insulin therapy, referred to our clinic with a 3-year history of multiple asymptomatic firm plaques disseminated on the upper and lower extremities. The clinical and histological findings proved the diagnosis of perforating necrobiosis lipoidica.

## 1. Introduction

Necrobiosis lipoidica diabeticorum is a rare idiopathic dermatological condition, commonly seen in women and frequently associates with diabetes. The perforating variant of disease in which necrotic collagen eliminates via transfollicular perforations is very seldom.

We present a case of type II diabetes patient with disseminated perforating necrobiosis lipoidica (PNL). According to our knowledge this is one of very few cases published in the literature.

## 2. Case Report

A 42-year-old Caucasian female patient suffering from long-term diabetes mellitus type II, controlled with rapid and retard acting insulin, was referred to our clinic. She complained of multiple asymptomatic firm plaques on the upper and lower extremities which enlarged peripherally and formed dark-brown centrally depressed plaques that appeared about 3 years ago. There was no history of trauma or operative interventions on the involved areas. One year later multiple reddish papules appeared on the upper extremities and slowly enlarged formatting indurated plaques.

**Figure 1:**
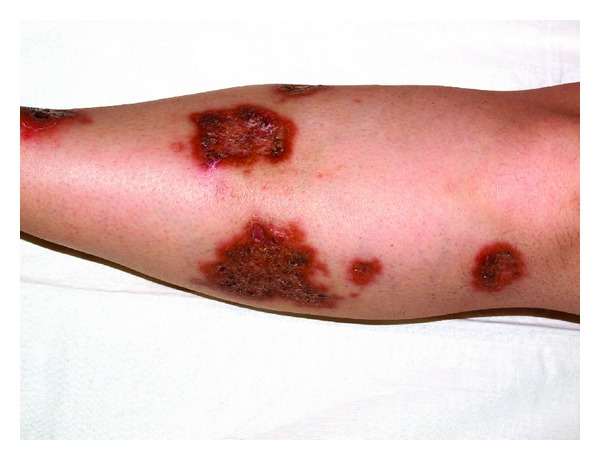
Infiltrated circinate plaques, erythematous border, and comedo-like papules in the center on the lower extremities of a 42-year-old diabetic patient.

**Figure 2:**
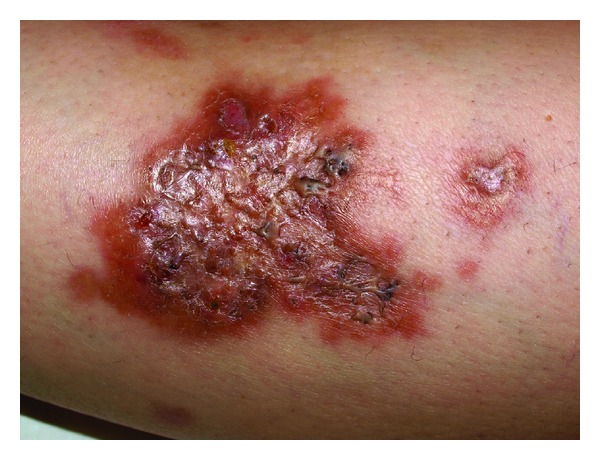
Close view of an irregularly shaped brown-yellow plaque with comedo-like papules.

The clinical examination revealed infiltrated plaques disseminated on the extensor surfaces of the upper and lower extremities. The lesions on the lower extremity were presented by brown-yellow plaques with different sizes, irregular shape, sharp slightly elevated border, and atrophic center focally studded with comedo-like papules (Figures 1 and 2). Some of the lesions are surrounded with erythematous halo and were painful at pressure. 

Systemic examination showed no diabetic retinopathy or neuropathy; arterial hypertension was controlled with propranolol with only single measurement of RR 150/90 mm/Hg at admission.

Laboratory investigations however revealed moderate anemia of 85 g/L and ketone bodies in urine. Blood sugar levels were fasting 4.2 mmol/L and postprandial 16.2 mmol/L. The patient was consulted with an endocrinologist, and insulin treatment was corrected.

**Figure 3:**
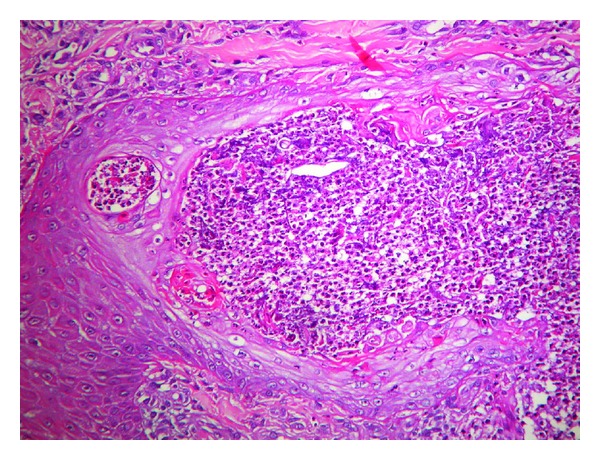
Histological examination demonstrated degenerated collagen and fibrosis with a peripheral lymphohistiocytic infiltrate in dermis (haematoxylin and eosin, original magnification ×200).

**Figure 4:**
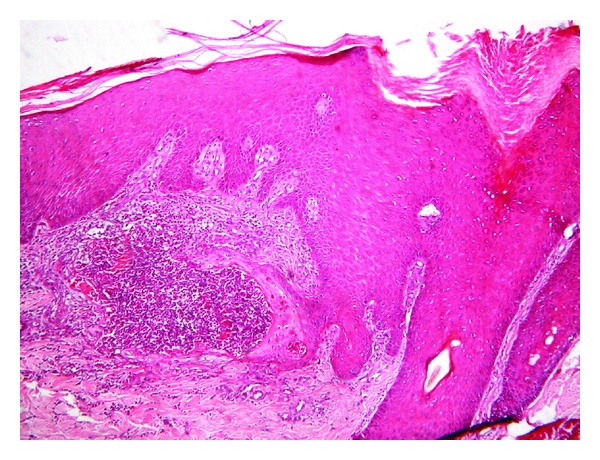
Histology demonstrates transepidermal elimination of necrotic material (haematoxylin and eosin, original magnification ×100).

The histological examination demonstrated degenerated collagen and fibrosis with peripheral lymphohistiocytic infiltrate (Figure 3) and transepidermal elimination of necrotic material (Figure 4).

The differential diagnosis included granuloma annulare, sarcoidosis, necrobiotic xanthogranuloma, lichen sclerosus et atrophicus, Darier-Roussy sarcoid, and erythema induratum of Bazin; however the clinical and histological findings were compatible with disseminated perforating necrobiosis lipoidica diabeticorum.

Initiated treatment with topical steroids was ineffective. Intralesional corticosteroid therapy was discussed but not started as the patient was lost to followup.

## 3. Discussion

Necrobiosis lipoidica diabeticorum was initially described by Oppenheim in 1929 [1], who termed it *dermatitis atrophicans lipoidica*. The perforating variant was presented by Parra in 1977, who published 3 clinical cases of transfollicular elimination of degenerated collagen presenting clinically as hyperkeratotic plugs on the surface of the plaques [2]. 

PNL belongs to the group of perforating disorders with transfollicular and transepidermal elimination of degenerated collagen or elastin as *perforating folliculitis*, *reactive perforating collagenosis*, *elastosis perforans serpiginosa, perforating pseudoxanthoma elasticum, and hyperkeratosis follicularis et parafollicularis in cutem penetrans*. Classification of these disorders is controversial; many physicians now recognizing three main perforating disorders: reactive perforating collagenosis, elastosis perforans serpiginosa, and acquired perforating dermatosis [3]. The common pathophysiological process in these disorders is the transepidermal elimination of dermal substances, predominantly collagen, keratin, or thickened elastic fibers [4]. Carter and Constantine are the first who postulate that in perforating diseases the keratinization focally occurs at the epidermal basilar layer, instead of *stratum corneum* as in normal epidermis [5, 6]. This causes a host inflammatory response, and keratin, cellular material, and connective tissue are being forced out of the skin through the epidermis [7]. Alteration of dermal connective tissue may also be a cause of inflammatory response. The clinical expression is typical: primary perforating disorders appear as hyperkeratotic, eroded papules, while secondary ones consisted of smooth plaques with centered keratotic papules and peripherally comedonal plugs [8]. The phenomenon of transfollicular and transepidermal elimination of degenerated collagen, elastic fibers, and necrotic tissue in necrobiosis lipoidica diabeticorum is rare, with few cases reported in the literature [8–12]. 

PNL associates in about 90% of cases with diabetes mellitus; however it is unrelated to the glucose control [13]. Our patient obviously had no good control of her diabetes. The review of the literature shows that PNL associates with diabetes type I in adolescents [11, 12] as well as type II in adult females 40–60 years [8, 9], as it was in our patient. The disease has a typically chronic course with the tendency of progression and scarring. Perforating necrobiosis lipoidica characterizes with appearance of one or multiple firm plaques with well-demarked borders. The presence of multiple perforating lesions, like those observed in our patient, is very seldom [9, 14]. Lesions affect more frequently lower extremities, less often arms, forearms, and trunk and seldom appeared on hands, fingers, face, and scalp [15]. Clinical efflorescence comprises of hyperpigmented plaques with central atrophy or hyperkeratotic papules and multiple comedo-like plugs in periphery [8]. These findings correspond histologically to the transepidermal elimination of degenerated connective tissue and are not characteristic for the classic type of necrobiosis lipoidica.

There are two histopathological types of necrobiosis lipoidica—a necrobiotic and a granulomatous one. The diabetic variation expresses necrobiotic changes, whereas in the nondiabetic form the granulomatous changes are present. In the necrobiotic type a necrobiosis of the collagen is present in the deep dermis with deposit of mucin and mixed inflammatory infiltrate consisting of lymphocytes, plasmocytes, histiocytes, fibroblasts, and epithelioid cells [16]. The chronic mechanical trauma may cause ulceration and scarring, which latter, as a complication, may contribute to the development of skin cancer [17]. Presently Mazzochi et al. described the appearance of perforating necrobiosis lipoidica at the site of healed herpes zoster, classifying it as the “Wolf's isotopic response” [18].

The local and systemic treatment often gives unsatisfactory results. In some cases the surgical therapy is useful, however in other it could provoke the Köbner phenomenon. The local therapy consists of the application of local corticosteroid [8], bovine collagen under occlusion [19], local retinoid and PUVA, photodynamic therapy [20], tacrolimus, intralesional corticosteroids, intralesional TNF-*α*, and perilesional application of heparin. The systemic therapy includes systemic corticosteroids, NSAID with dipyridamole, cytostatics, and thalidomide.

In conclusion, we present a rare case of a 42-year-old patient with disseminated perforating necrobiosis lipoidica diabeticorum. Although the disease has benign course, the therapeutic resistance and uncontrolled diabetes may cause serious outcome.
